# Antigen Presentation by Vascular Cells

**DOI:** 10.3389/fimmu.2017.01907

**Published:** 2017-12-22

**Authors:** Jordan S. Pober, Jonathan Merola, Rebecca Liu, Thomas D. Manes

**Affiliations:** ^1^Department of Immunobiology, Yale School of Medicine, New Haven, CT, United States; ^2^Department of Surgery, Yale School of Medicine, New Haven, CT, United States

**Keywords:** endothelial cells, pericytes, smooth muscle cells, effector memory T cells, regulatory T cells, transendothelial migration

## Abstract

Antigen presentation by cells of the vessel wall may initiate rapid and localized memory immune responses in peripheral tissues. Peptide antigens displayed on major histocompatibility complex (MHC) molecules on the surface of endothelial cells (ECs) can be recognized by T cell receptors on circulating effector memory T cells (T_EM_), triggering both transendothelial migration and activation. The array of co-stimulatory receptors, adhesion molecules, and cytokines expressed by ECs serves to modulate T cell activation responses. While the effects of these interactions vary among species, vascular beds, and vascular segments within the same tissue, they are capable of triggering allograft rejection without direct involvement of professional antigen-presenting cells and may play a similar role in host defense against infections and in autoimmunity. Once across the endothelium, extravasating T_EM_ then contact mural cells of the vessel wall, including pericytes or vascular smooth muscle cells, which may also present antigens and provide signals that further regulate T cell responses. Collectively, these interactions provide an unexplored opportunity in which targeting of vascular cells can be used to modulate immune responses. In organ transplantation, targeting ECs with siRNA to reduce expression of MHC molecules may additionally mitigate perioperative injuries by preformed alloantibodies, further reducing the risk of graft rejection. Similarly, genetic manipulation of vascular cells to minimize antigen-dependent responses can be used to increase perfusion of tissue engineered organs without triggering rejection.

## Introduction

Primary adaptive immune responses are initiated when foreign antigens are presented to naïve T cells by “professional” myeloid antigen-presenting cells (APCs), such as dendritic cells (DCs) residing in secondary lymphoid organs. In response to antigen recognition, naïve T cells both expand and differentiate into effector T cells and various memory T cell subsets. Memory T cells persist long after antigen clearance, and in response to reappearance of the same antigen, rapidly initiate secondary (memory) responses directly at sites where antigen has reappeared (1). Memory T cells may reside within the tissues (T_RM_) or circulate as effector memory T cells (T_EM_) that are recruited to peripheral tissues following “activation” of the local microvasculature induced by inflammatory cytokines, pathogen-associated or damage-associated molecular patterns (2). Activated endothelial cells (ECs) lining the local vasculature express adhesion molecules and elaborate chemokines that capture and promote transmigration of T_EM_ (3, 4). Alternatively, T_EM_ may be directly recruited by antigens presented by ECs (5). Extravasating T_EM_ must then interact with the mural cells that support the ECs, namely pericytes (PCs) in microvessels or vascular smooth muscle cells (VSMCs) in larger vessels, that may also present antigens that modulate T_EM_ responses (6, 7). Here, we review the topic of antigen presentation by these vascular cell types.

## Antigen Presentation by ECs

### Major Histocompatibility Complex (MHC) Molecules

The capacity of cells to form and display peptide antigens complexed to MHC molecules, class I to CD8+ T lymphocytes and class II to CD4+ T lymphocytes, is a prerequisite for antigen presentation. Peptide antigens presented by ECs may be self-derived, though the same peptide–MHC complex may be tolerogenic or pro-inflammatory in response to the milieu in which lymphocyte encounter occurs (8). Microvascular ECs in humans and most other mammals basally express both class I and class II MHC molecules *in vivo* (9, 10). However, the abundance of MHC expression by ECs differs among vascular segments and can vary in response to environmental signals (11). Unlike other mammals, mouse and rat ECs do not basally express class II molecules *in vivo* (12, 13), a significant consideration when extrapolating results from murine models to human pathology.

Human ECs reduce their level of class I and ablate class II MHC expression in cell culture. Class I MHC expression can be restored by type 1 interferons (IFN-α or -β), type 2 interferon (IFN-γ), or tumor necrosis factors (both TNF-α or lymphotoxin-α), whereas class II MHC is induced only by IFN-γ (14, 15). *In vivo*, basal expression of class I MHC is lost on arterial ECs in knockout mice lacking IFN-γ or the IFN-γ receptor (16). In canines, treatment with cyclosporine to inhibit cytokine production similarly results in loss of basal class II MHC molecule expression on arterial ECs (17). The expression of both classes of MHC molecules requires concomitant expression of several proteins required for peptide generation and loading. In cultured human ECs, these proteins are coordinately regulated by the same cytokines that induce class I and class II MHC (18, 19).

### Co-stimulatory Signals

Effective antigen presentation to T cells by an APC additionally requires antigen-independent signals delivered by cell surface co-stimulators that engage T cell counter-receptors and signal to augment and complement T cell receptor (TCR) signals (20, 21). The specific co-stimulators required for effective T cell activation differ for naïve and memory T cells and among memory T cell subsets. The co-stimulators B7.1 (CD80) and B7.2 (CD86) that engage T cell CD28 appear indispensable for activation of naïve T cells though expression of these molecules on ECs has been controversial. While some reports note the presence of B7.1 and B7.2 in human EC cultures (22–27), several other reports were unable to produce these findings (28–32). The absence of these molecules is consistent with the inability of human ECs to activate allogeneic naïve T cells (30, 33). However, cultured human ECs can activate allogeneic memory T cells using other co-stimulators and may do so with comparable or greater proficiency than professional myeloid APCs (30, 33, 34). The most potent co-stimulator of memory T cells on human ECs is lymphocyte function-associated antigen (LFA)-3 (CD58), which engages CD2, a counter-receptor expressed more highly on T_EM_ than on naïve T cells (33). Other important co-stimulators expressed basally or inducibly on human ECs which engage cognate receptors on activated memory T cells are summarized in Table 1.

**Table 1:** Cell surface proteins mediating antigen presentation by endothelial cells (ECs) to T_EM_.

EC molecule	T_EM_ counter-receptor	Function	Reference
Major histocompatibility complex (MHC) class I	T cell receptor (TCR), CD8	Activation	(9)
MHC class II	TCR, CD4	Activation	(10)
B7.1 (CD80), B7.2 (CD86)^a^	CD28	Costimulation	(22–27)
LFA-3 (CD58)	CD2	Costimulation	(33)
ICOS-L (CD275)	ICOS (CD278), CD28^b^	Costimulation	(33)
Ox40-L (CD252)	Ox40 (CD134)	Costimulation	(33)
4-1BB-L (CD137L)	4-1BB (CD137)	Costimulation	(33)
CD40L (CD154)	CD40	Costimulation	(33)
CD40	CD40L (CD154)	*Unknown*	(23)
PD-L1 (CD274), PD-L2 (CD273)	PD-1	Inhibition	(35)
B7.1 (CD80), B7.2 (CD86)^a^	CTLA-4	Inhibition	(22–27)
CD155	TIGIT	Inhibition?	(36)
Galectin-9, CEACAM-1	TIM3	Inhibition?	(37, 38)
*Unknown*	LAG3 (CD223)	Inhibition?	(37)
E-Selectin (CD62E)	ESL-1, ESL-2, CLA-1	Tethering and rolling	(3)
P-Selectin (CD62P)	PSGL-1	Tethering and rolling	(39)
ICAM-1	LFA-1 (CD11a/CD18)	Firm adhesion, diapedesis, costimulation	(3, 40, 41)
VCAM-1	VLA-4	Rolling, firm adhesion, diapedesis^c^	(42)
PECAM-1 (CD31)^c^	*Unknown*	Diapedesis	(43)
MIC2 (CD99)^c^	MIC2 (CD99)	Diapedesis	(4)
PVR (CD155), Nectin-2 (CD112)^c^	Tactile (CD96), DNAM (CD226)	Diapedesis	(4)

*^a^Not consistently found on human ECs*.

*^b^Humans only*.

*^c^CD4 T_EM_ only*.

Endothelial cells may also display signals that dampen T_EM_ activation, referred to as negative co-stimulators or checkpoint inhibitors (Table 1). Counter-receptors for negative co-stimulatory are expressed predominantly on activated T cells. CTLA-4 (CD152), the first described inhibitory receptor, binds B7.1 and B7.2 with higher affinity than does CD28 (44, 45), but as noted above, these proteins have not been consistently observed on human ECs. IFN-γ induces mouse ECs to express PD-L1 (CD274) and human ECs to express both PD-L1 and PD-L2 (CD273) (35, 46), negative co-stimulators that engage PD-1 (CD279) expressed on activated T_EM_. Other T cell inhibitory receptors include TIGIT, TIM3, and LAG3 (37), but it is not yet known if any EC proteins deliver negative co-stimulatory signals through these molecules.

### Adhesion Molecules

In addition to peptide–MHC and co-stimulatory recognition, effective activation of T cells requires stable attachment to the APCs for several minutes. Molecules that contribute to T cell adhesion to ECs are summarized in Table 1. At sites of inflammation, initial tethering and subsequent rolling (propelled by flowing blood) of T cells is largely mediated by interactions of selectins expressed on activated ECs with glycosylated ligands on T lymphocytes. T cell recognition of chemokines displayed on the EC lumen triggers T cell spreading, firm attachment and migration to EC junctions. These higher affinity interactions are mediated by T cell integrins (47). Notably, T_EM_ express high levels of LFA-1 (CD11a/CD18) and very late activation antigen (VLA)-4 (CD49d/CD29), two key integrins that specifically bind to proteins that are highly expressed on cytokine-activated human ECs, namely intercellular adhesion molecule (ICAM)-1 and vascular cell adhesion molecule (VCAM)-1, respectively (48). High affinity integrin binding requires both TCR signals and “tugging” of the integrin as the T cell is being pushed by flowing blood (49). In their high affinity state, these integrins enable T_EM_ to firmly attach to activated microvascular ECs resisting detachment by blood flow. Engagement of LFA-1 integrin on T lymphocytes can stabilize mRNA transcripts of pro-inflammatory genes, implicating a dual role for these molecules in both T cell activation and adhesion (40, 41). Other human EC proteins engage counter receptors on T_EM_ during the process of transendothelial migration (diapedesis), though whether these molecules influence TCR signaling is currently unknown.

### Cytokine Signals

Cytokines produced by an activated T cell, by its APC, or by a bystander cell, provide a third class of signal that can influence the magnitude of a T cell response (50). ECs can be stimulated to release a number of active cytokines, but secreted molecules will be rapidly removed by blood flow. The effects of flow may be overcome by displaying cytokines bound to the EC surface. Many chemokines bound to the EC surface *via* the glycocalyx or *via* non-signaling receptors can activate attached leukocytes (51, 52). Although its method of attachment is unknown, interleukin (IL)-1α can be displayed on the plasma membrane of human ECs and signal to T cells to increase their activation (53).

### Mechanisms of Allopresentation

Because T_EM_ specific for a given protein antigen are rare, even after expansion from naïve precursors, human responses are typically evaluated *in vitro* shortly after vaccination or by using polyclonal activators, superantigens, or non-self alloantigens. Alloantigen responses arise from cross-reacting T_EM_ generated from naïve T cells activated in response to prior infections (54). Cultured human ECs can activate resting alloreactive CD8+ T_EM_, measured as cytokine production or proliferation (34), and the latter response is enhanced by addition of exogenous IL-2, normally provided *in vivo* by activated CD4+ T_EM_ (55). Although exogenous protein antigens are typically presented to CD4+ T cells as peptides bound to class II MHC molecules, professional APCs can “cross-present” to CD8+ T cells by loading such peptides on to class I MHC molecules. Mouse ECs also “cross-present” antigen (56–58), but this has not been demonstrated in humans. However, human ECs may acquire intact class I MHC peptide complexes from other cells and then present these to T cells, a pathway of alloantigen presentation referred to as “cross-dressing” or “semi-direct presentation” (59).

Cultured human ECs can stimulate alloreactive CD8+ T_CM_ or T_EM_, but not naïve T cells, to differentiate into cytotoxic T lymphocytes (CTL) (60). Interestingly, many of these CTL appear specific for ECs that share class I MHC alleles with the ECs used for stimulation but will not lyse B cells expressing the same alleles (61). This may represent a requirement for EC-derived peptides, differences in peptide processing, or a requirement of EC-specific adhesive ligands or co-stimulators. Cultured human ECs, induced to express class II MHC molecules by pretreatment with IFN-γ, effectively activate CD4+ T_CM_ and T_EM_ (34). In contrast, cultured mouse ECs, similarly induced to express class II MHC molecules, cannot activate resting CD4+ T cells to exhibit effector functions, but have been reported to activate (or induce) CD4+ T cells with regulatory functions (62). These differences are unexplained, but again raise caution when extrapolating observations from mouse experiments to human settings. In contrast, mouse ECs, which typically express B7.1, can activate naïve allogeneic CD8+ T cells in culture (63) and initiate allograft rejection *in vivo* in the absence of graft-derived professional APCs (64). In the case of allografts, T_RM_ are graft-derived so antigen presentation to circulating host T cells by graft ECs or DCs is required to initiate chemokine-mediated T cell recruitment and vigorous rejection (65).

### Lymphocyte Transendothelial Migration

Cell culture experiments using superantigen have revealed that both CD4+ and CD8+ T_EM_ that recognize antigens on the apical surface of human microvascular ECs are induced to transmigrate through the EC barrier (66). Morphologically, the initial T cell response to antigen recognition on ECs appears similar to that which occurs during antigen presentation by professional APCs. T_EM_ “round up,” move their microtubule organizing center (MTOC) and cytosolic granules to the region between the nucleus and the EC surface (67). Despite flow, TCR-engaged T_EM_ remain attached to the EC for 30 min or more, i.e., sufficiently long enough for activation to occur. The attached T_EM_ then extend a blunt cytosolic protrusion between adjacent ECs. Granules and the MTOC move into the protruding leading edge and the nucleus follows. Events in TCR-triggered transendothelial migration of human T_EM_ are distinct from chemokine-mediated transendothelial migration in which T_EM_ initially flatten out rather than rounding up and in which the MTOC and granules follow the nucleus between adjacent ECs in a trailing uropod rather than preceding the nucleus in a blunt protrusion. During TCR-triggered transendothelial migration, CD4+ T_EM_ discharge granules, releasing granzyme A, the proteolytic activity of which is required for effective transmigration (67). In contrast, CD8+ T_EM_ transits through the EC monolayer without discharging their granules, which contain granzyme B and perforin in addition to granzyme A (66). The basis for this difference in granule release is unknown but protects EC from lysis. During transendothelial migration, CD4+ T_EM_ also mobilize and interact with EC junctional proteins present in the lateral border recycling compartment, including PECAM-1 (CD31), CD99, polio virus receptor (CD155), and nectin-2 (CD112) (4), whereas CD8+ T_EM_ bypass this requirement. Interestingly, mice lacking endothelial PECAM-1 are still able to recruit CD8+ T_EM_ and actually appear to do so more efficiently than wild-type mice (68). It is unclear if mouse ECs use antigen presentation to recruit CD4+ T_EM_. TCR-induced human T_EM_ migration also involves reorganization of the actomyosin cytoskeleton, which is activated by TCR signaling through Vav and Rac, whereas chemokine-induced transmigration bypasses Vav and uses cdc42 instead of Rac to activate the cytoskeleton (69). While it is difficult to confirm that the same processes occur during human immune response *in vivo*, experiments in mice have demonstrated Vav-dependent TCR-based recruitment (70) as well as a distinction between TCR-and chemokine-initiated T cell recruitment based on only the latter showing pertussis toxin sensitivity (65).

### Activation of Regulatory T Cells

Presentation of self-antigen linked to class II MHC molecules by ECs may be involved in the recruitment of CD4+ T regulatory cells (T_REG_) to peripheral tissues (71). Under certain conditions, antigen presentation by human ECs may directly favor T_REG_ activation. Specifically, unlike T_EM_ activation, T_REG_ proliferation is not inhibited by pretreatment of human EC with the mTOR inhibitor, rapamycin, thereby favoring T_REG_ expansion (72). Blockade of endothelial secreted IL-6 may promote the conversion of T_H_17 memory cells into T_REG_ (73). It is unknown if recruitment of the recently described CD4+ T peripheral helper cell (T_PH_) subset that can activate B cell responses in peripheral tissues can be recruited by EC antigen presentation (74). Finally, some specialized ECs may use antigen presentation to specifically tolerize T cells and inhibit immune responses. This has been described both for hepatic sinusoidal ECs and for lymphatic ECs in the mouse (75–77). However, the mechanisms of tolerance induction employed by these cells are not well understood, nor is it clear if the same mechanisms apply to human ECs lining such vessels.

## Antigen Presentation by PCs and VSMCs

### Antigen Presentation by VSMCs

T_EM_ that enter tissues or the vessel wall (in the case of large blood vessels), will then encounter mural cells: PCs in microvessels or VSMCs in larger vessels. VSMCs express low levels of class I but not class II MHC molecules *in situ;* the abundance of MHC molecule expression by PCs *in situ* is unclear as resolving ECs and PCs by light microscopy is challenging. Under culture conditions human PCs or VSMCs, like cultured human ECs, similarly express low levels of class I and no detectable class II MHC but readily upregulate both class I and class II MHC molecules to levels comparable to those expressed on ECs in response to IFN-γ (6, 78). Moreover, following IFN-γ treatment, both mural cell types appear able to present non-self alleles of class II MHC molecules to resting allogeneic CD4+ T cells, inducing expression of activation antigens on the T cell, such as CD69 and CD25. CD4+ T cells already activated by culture with IFN-γ-treated ECs, derived from the same donor as the VSMCs, can proliferate in response to IFN-γ-treated VSMCs (78). However, resting T_EM_ directly isolated from peripheral blood will not proliferate in these cultures, but, such T cells cultured with VSMCs can be subsequently activated by culture with ECs from the same donor (i.e., they are not anergized). Failure of resting T cells to proliferate in response to allogeneic VSMCs may be attributed both to the expression of indolamine 2,3-dioxygenase (IDO), an enzyme that degrades tryptophan required for T cell anabolism and cell proliferation, and to the absence of ICOS-L, a critical co-stimulator (79, 80). In contrast, ECs readily express ICOS-L and significantly less IDO than VSMCs.

### Antigen Presentation by PCs

Less has been reported about immunoregulation by human PCs. IFN-γ-treated PCs express negative co-stimulatory receptors PD-L1 and PD-L2 at a higher level than do ECs. The role of PD-L1 or PD-L2 in inhibiting the activation of resting T cells, which typically lack PD-1, is largely unknown. Like VSMCs, IFN-γ-treated PCs express high levels of IDO that contributes to T cell inhibition (6). Importantly, CD4+ T cells cultured with allogeneic PCs do become clonally anergic, i.e., they are unable to subsequently respond to ECs from the same donor as the PCs. It is unknown how much of the inhibitory activities of PCs or VSMCs are induced by IFN-γ, utilized in these experiments to upregulate class II MHC molecules on the mural cells. There are likely to be differences among PCs (and VSMCs) from different species and among the mural cells found in different vascular beds within the same species. Cultured mouse PCs can express PD-L1 and are also immunoregulatory (81), but it has not been shown if mouse PCs present antigen. Despite the limited number of studies, the general conclusion is that mural cells present antigens in a manner that dampens (“regulate”) T cell responses that are initiated by EC antigen presentation.

## Therapeutic Targeting of Antigen Presentation by Vascular Cells

The most compelling data regarding the antigen-presenting function of ECs has come from transplantation. As noted earlier, this may be explained by the fact that the T_RM_ population within an allograft is derived from the donor and will, therefore, not respond to donor antigens as non-self. Consequently, the most relevant memory T cell population in transplant immunology is T_EM_ which preferentially respond to antigens presented in the vascular lumen. While genetic manipulation of human organs for transplantation is not an immediate prospect, transient knockdown of gene expression by siRNA or anti-sense oligonucleotides in graft cells is much closer to clinical application, and a particularly attractive approach is to deliver a sustained source of siRNA to graft vessels *ex vivo*, prior to implantation, using hydrolyzable nanoparticle carriers (81). Relevant targets could include MHC molecules or co-stimulators such as LFA-3 that promote T cell activation or alternatively induction of negative co-stimulatory molecules in ECs or mural cells. Interestingly, rapamycin, which also could be delivered in nanoparticle carriers *ex vivo*, has been found to induce both PD-L1 and PD-L2 on ECs independently of IFN-γ, abrogating their capacity to activate T_EM_ while retaining the capacity to activate T_REG_ (72). The roles that vascular cells perform as APCs represent a largely untapped therapeutic target in these pathologic settings.

Human ECs have been implicated as APCs in other chronic disease states. For example, in type I diabetes, EC antigen presentation triggers T cell homing leading to islet cell destruction (27, 56), and in cerebral malaria, presentation of parasite antigens may activate CD8 effector cells leading to local edema and inflammation in the central nervous system (82). In both autoimmunity and infection, however, the role of T_EM_ may be redundant with that of T_RM_, and may potentially limit the effects of blocking antigen presentation by ECs, though the effects of doing so remain to be investigated.

## Author Contributions

JSP authored the manuscript. JM, RL, and TM provided critical revisions to the manuscript.

## Conflict of Interest Statement

The authors declare that the research was conducted in the absence of any commercial or financial relationships that could be construed as a potential conflict of interest.
